# High-throughput metal susceptibility testing of microbial biofilms

**DOI:** 10.1186/1471-2180-5-53

**Published:** 2005-10-03

**Authors:** Joe J Harrison, Raymond J Turner, Howard Ceri

**Affiliations:** 1 Department of Biological Sciences, University of Calgary, 2500 University Drive N.W., Calgary, Alberta, Canada T2N 1N4; 2 Biofilm Research Group, University of Calgary, 2500 University Drive N.W., Calgary, Alberta, Canada T2N 1N4

## Abstract

**Background:**

Microbial biofilms exist all over the natural world, a distribution that is paralleled by metal cations and oxyanions. Despite this reality, very few studies have examined how biofilms withstand exposure to these toxic compounds. This article describes a batch culture technique for biofilm and planktonic cell metal susceptibility testing using the MBEC assay. This device is compatible with standard 96-well microtiter plate technology. As part of this method, a two part, metal specific neutralization protocol is summarized. This procedure minimizes residual biological toxicity arising from the carry-over of metals from challenge to recovery media. Neutralization consists of treating cultures with a chemical compound known to react with or to chelate the metal. Treated cultures are plated onto rich agar to allow metal complexes to diffuse into the recovery medium while bacteria remain on top to recover. Two difficulties associated with metal susceptibility testing were the focus of two applications of this technique. First, assays were calibrated to allow comparisons of the susceptibility of different organisms to metals. Second, the effects of exposure time and growth medium composition on the susceptibility of *E. coli *JM109 biofilms to metals were investigated.

**Results:**

This high-throughput method generated 96-statistically equivalent biofilms in a single device and thus allowed for comparative and combinatorial experiments of media, microbial strains, exposure times and metals. By adjusting growth conditions, it was possible to examine biofilms of different microorganisms that had similar cell densities. In one example, *Pseudomonas aeruginosa *ATCC 27853 was up to 80 times more resistant to heavy metalloid oxyanions than *Escherichia coli *TG1. Further, biofilms were up to 133 times more tolerant to tellurite (TeO_3_^2-^) than corresponding planktonic cultures. Regardless of the growth medium, the tolerance of biofilm and planktonic cell *E. coli *JM109 to metals was time-dependent.

**Conclusion:**

This method results in accurate, easily reproducible comparisons between the susceptibility of planktonic cells and biofilms to metals. Further, it was possible to make direct comparisons of the ability of different microbial strains to withstand metal toxicity. The data presented here also indicate that exposure time is an important variable in metal susceptibility testing of bacteria.

## Background

Determination of the minimum inhibitory concentration (MIC), based on antimicrobial activity against planktonic organisms, is the standard assay for susceptibility testing. Biofilms, which present with distinct physiology compared to planktonic cells, are infamous for their ability to withstand a wide range of antimicrobials, including metals [1-4]. Despite the ubiquitous distribution of metals and the predominance of microbial biofilms in the environment and in device-associated infections, very few studies have comparatively examined biofilm susceptibility to metals relative to planktonic cells. The scarcity of data in this regard may be attributable to the existing methods used to grow biofilms, which typically include contamination prone flow systems. Metal susceptibility testing also entails challenges not encountered with antibiotics. This includes complexation of metals with components of growth media, inorganic precipitation, reduction reactions, and carry-over of the metal to the recovery medium.

A recently developed, high-throughput approach to antibiotic and biocide susceptibility testing of microbial biofilms is the Calgary Biofilm Device [5,6] (commercially available as the MBEC-high throughput (HTP) assay, MBEC Bioproducts Inc., Edmonton, Alberta, Canada [7]). This batch culture method of biofilm and planktonic cell susceptibility testing provides three internally consistent, comparative measurements from a single experiment: 1) the planktonic minimum inhibitory concentration (MIC), 2) the planktonic minimum bactericidal concentration (MBC), and 3) the minimum biofilm eradication concentration (MBEC). The MBEC assay is not prone to leakage and contamination since it is manipulated in a Laminar flow hood.

The present study was rooted in two principle aims. The first aim was to develop a method of high-throughput metal susceptibility testing of biofilms using the MBEC assay. As part of this goal, a metal specific neutralizing regime was employed to reduce the biological toxicity of many different metal cations and oxyanions *in vitro*. This procedure allowed for comparisons between the susceptibility of planktonic cells and biofilms to metals (between different strains and/or microbial species), and provides a significant modification of the procedure originally reported by Ceri *et al*. for antibiotic susceptibility testing [5,6]. Also presented here is quality control data for the MBEC technique that has not been published elsewhere.

The second aim was to apply this method to examine variables that may influence measurements of metal susceptibility. A common dilemma in comparative studies of different bacterial strains is the ability of each strain to form biofilms. In simple terms, the ratio of bacterial cells (i.e. chemically reactive targets) to metal ions may influence the determination of susceptibility. To address this, biofilm growth of different bacterial species was calibrated to allow relative comparisons of susceptibility between biofilms with similar cell densities. Here, the relative differences in *E. coli *and *P. aeruginosa *biofilm susceptibility to the heavy metalloid oxyanions selenite (SeO_3_^2-^) and tellurite (TeO_3_^2-^) were examined. These compounds are highly toxic, water soluble pollutants that are spread into the environment in the form of industrial effluent [8,9]. *P. aeruginosa *was 80 times more resistant to heavy metalloids than *E. coli*.

Another potential obstacle to accurate measurements from metal susceptibility testing is exposure time. Studies of short duration metal exposure in minimal media have indicated that biofilms are highly tolerant to heavy metals [2,3], whereas long exposures in rich media resulted in complete elimination of the biofilm [3]. Logically, these differences may be based on differences in either exposure time or growth medium. Here, the effect of these variables on the measured tolerance of *E. coli *biofilms to metal cations was examined. Time-dependent trends in biofilm susceptibility to metals were identified that were not altered by changing the medium in which bacteria were grown or challenged. Exposure time is thus an important consideration in the design of studies centred on biofilm tolerance to metals.

## Results

### Biofilm growth

Biofilms of *Pseudomonas aeruginosa *ATCC 27853, *E. coli *TG1, and *E. coli *JM109 were grown to an overall mean density of 6.6 ± 0.5, 6.7 ± 0.3, and 6.4 ± 0.4 log_10 _cfu peg^-1 ^(respectively) in Luria-Bertani medium enriched with vitamin B1 (LB + B1). This corresponded to 9.5 and 24 h of incubation at 35°C for *P. aeruginosa *and *E. coli*, respectively. When grown in minimal salts vitamins glucose (MSVG) medium for 24 h at 35°C, biofilms of *E. coli *JM109 reached a mean cell density of 5.02 ± 0.55 log_10 _cfu peg^-1^. Mean and standard deviation calculations for cell densities were based on pooled data from all growth controls performed (i.e. from 36 to 59 replicates each).

**Figure 1:**
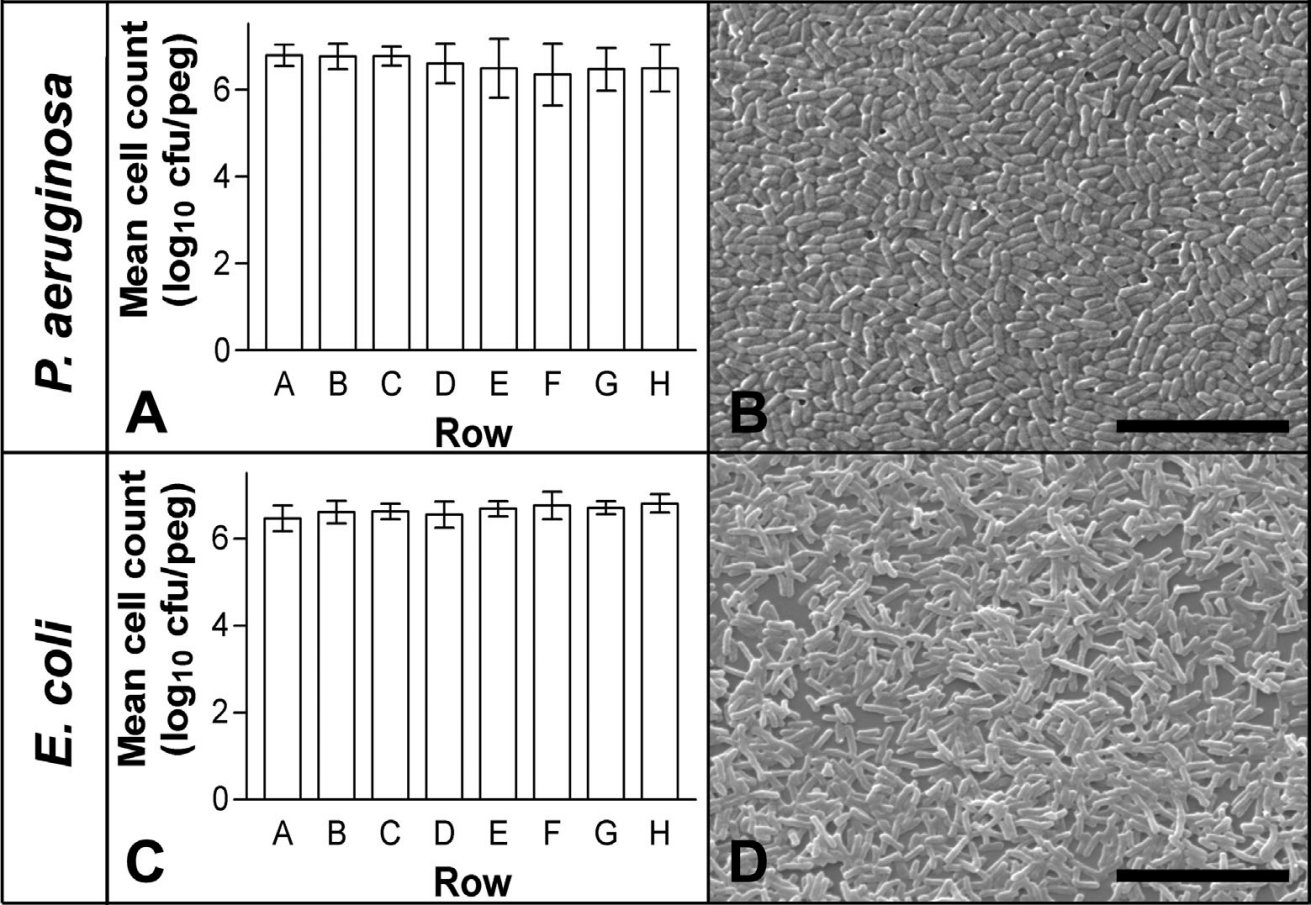
**Growth of bacterial biofilms in the MBEC assay**. (A) Mean cell density of *Pseudomonas aeruginosa *ATCC 27853 biofilms on the pegs in different rows of the MBEC assay. Each value is expressed as the mean and standard deviation of 4 to 6 trials. There is no significant difference between cell density of biofilms in the different rows (*p *= 0.842 using one-way ANOVA). (B) SEM photomicrograph of a *P. aeruginosa *biofilm on the peg surface. (C) Mean cell density of *Escherichia coli *TG1 on the pegs in different rows of the MBEC assay. Each value is expressed as the mean and standard deviation of 4 to 6 trials. There is no significant difference between cell density of biofilms in the different rows (*p *= 0.274 using one-way ANOVA). (D) SEM photomicrograph of an *E. coli *biofilm on the peg surface. The bar represents 5 μm.

Mean viable cell counts and standard deviation (SD) for *P. aeruginosa *ATCC 27853 and *E. coli *TG1 on the pegs of each row of the MBEC™-HTP assay are presented in (Fig. 1a and 1c, respectively, 4 to 6 replicates each). The cell counts for each row were pooled and compared using one-way analysis of variance (ANOVA). The cell density of biofilms grown on the different rows of pegs in the MBEC™-HTP assay were statistically equivalent (*p *= 0.842 for *P. aeruginosa*; *p *= 0.274 for *E. coli*). *E. coli *JM109 also formed statistically equivalent biofilms across the different rows of pegs. This was similar to *E. coli *TG1, and thus the data is not presented here.

To allow for a valid comparison of susceptibility data, the biofilm cell counts for *P. aeruginosa *ATCC 27853 and *E. coli *TG1 were compared using a Mann-Whitney U-test. The biofilm cell density of these two strains were statistically equivalent (*p *= 0.209). Under the growth conditions reported, *E. coli *JM109 formed biofilms with significantly less cell density than the other 2 strains when compared using a Mann-Whitney U-test (*p *< 0.001).

### Microscopy

Biofilms were examined *in situ *using scanning electron microscopy. Photomicrographs of *P. aeruginosa *ATCC 27853 and *E. coli *TG1 are presented in (Fig. 1b and 1d, respectively). SEM pictures show the growth of surface-adherent bacteria in thin layers and mounds on the pegs of the MBEC™ device. These layers were estimated to be up to 10 μm in height in some areas. Biofilms heterogeneously covered the plastic surface. The biofilm growth of *E. coli *JM109 was similar to strain TG1, except with a slightly more sparse distribution across the peg surface (data not shown).

### Susceptibility of *E. coli *and *P. aeruginosa *to metalloid oxyanions

In this study and as an example, biofilms of *Escherichia coli *TG1 and *Pseudomonas aeruginosa *ATCC 27853 were assayed for susceptibility to selenite (SeO_3_^2-^) and tellurite (TeO_3_^2-^). These two organisms formed biofilms with statistically equivalent cell density when grown as described above. This allowed for a direct comparison between the two bacterial strains for relative levels of resistance to these heavy metalloid oxyanions.

For *E. coli *TG1 and *P. aeruginosa *ATCC 27853, the mean and standard deviation of MIC, MBC and MBEC values for SeO_3_^2- ^and TeO_3_^2- ^are summarized in Table 1. These calculations were based on 4 to 8 independent replicates each. With 4 h of exposure, biofilms were up to 133 times more tolerant to TeO_3_^2- ^than the corresponding planktonic cultures. With regards to planktonic cells (derived from the surface of the biofilms), *P. aeruginosa *was 80 times more resistant to tellurite than *E. coli*.

### Time-dependent susceptibility of *E. coli *biofilms to metal cations

Exposure time may be of pivotal importance as a controlled variable in the design of metal susceptibility assays. To directly address this problem, an array of 11 metal cations was chosen to represent groups 7B to 4A of the periodic table. Using the MBEC assay, the susceptibility of *E. coli *JM109 to these compounds was tested. *E. coli *JM109 has been a popular model microorganism for studies of metal resistance in bacteria. Biofilm and planktonic cell susceptibility at 2 and 24 h of exposure in both rich medium (LB + B1) and minimal medium (MSVG) was examined. A larger data set with a greater number of trials at 24 h of exposure in LB + B1 was used in this study to expand on an original report [10]. All other test conditions were previously unexamined. This data is presented in Tables 2 and 3, respectively.

In general, MIC, MBC and MBEC values were greater in rich medium than in minimal medium. This was probably due to the chelation of metal ions by phosphates and organic matter present in the growth medium. However, there were two trends that were invariant with regards to nutrient status: 1) In LB + B1, biofilms were 1.3 to 24 times more tolerant to metal cations than the corresponding planktonic cells when exposed for 2 h. Similarly, *E. coli *biofilms grown and tested in MSVG were 2.0 to 29 times more tolerant to metal cations with a similar exposure time. In combination with the susceptibility data for SeO_3_^2- ^and TeO_3_^2-^, this would suggest that with short exposures biofilms are highly tolerant to metal toxicity. 2) By 24 h of exposure, biofilm and planktonic cultures were eradicated at similar concentrations of metal cations in almost every instance. This occurred independently of the growth medium used for bacterial susceptibility testing.

Collectively, these data suggest that biofilm tolerance to metals is time-dependent. We note that (in general) MIC values did not change with exposure time using this method (data not shown).

## Discussion

This manuscript describes a high-throughput method for metal susceptibility testing of biofilms using the MBEC assay. This technique has the advantage that metal susceptibility testing may be done using a combinatorial approach. It is possible to employ different mean numbers of bacterial cells, alternate exposure times, various and diverse growth media formulations, as well as a broad range of metals (as well as other antimicrobial compounds). Many bacteria and yeasts, including *Staphylococcus aureus *[6,10], *Mycobacterium *spp. [11], *Candida *spp. (J.J. Harrison, H. Ceri and R.J. Turner, unpublished data), and *Burholderia cepacia *complex [12] are amenable to biofilm growth using the MBEC assay. Biofilms on the pegs of the MBEC device may be examined *in situ *using scanning electron microscopy, epifluorescent microscopy [13], and confocal laser-scanning microscopy (CLSM) [14].

As a quality control, a test for equivalent biofilm formation between rows of pegs in the MBEC device was performed. Biofilm cell density was statistically equivalent across the different rows of pegs for *E. coli *TG1, JM109 and *P. aeruginosa *ATCC 27853. This quality control needs to be a routine test for studies of biofilm susceptibility using this method. It is important to note that not all bacteria (particularly mucoidal isolates) are amenable to this method of growth (J.J. Harrison, H. Ceri and C. Stremick, unpublished data). If statistically equivalent biofilms are not generated using the MBEC trough format, it is possible to instead place the peg lid in a microtiter plate containing ~150 μl of inoculum in each well. This alternative system may be placed on a gyrorotary shaker to facilitate biofilm formation on the peg lid. In general, biofilms formed using a trough have a 5- to 10-fold greater cell density than those formed using the microtiter plate format (J.J. Harrison, H. Ceri and C. Stremick, unpublished data).

This procedure may be modified to discern biofilm and planktonic viable cell counts. This is included as an amendment with the online supplementary material. Viable cell counting is accomplished by serially diluting aliquots from the recovery and neutralization plates, which are subsequently plated onto agar and enumerated. A characteristic of the MBEC assay is that planktonic cultures are seeded by cells shed from the biofilm. Log-killing of biofilms may be calculated using this method, but as a consequence, log-killing of planktonic cells may not. However, this model does reflect the natural duality of the bacterial life cycle where a recalcitrant nidus of biofilm cells may survive metal exposure to shed planktonic cells back into the surroundings. Antibiotic MIC values obtained from this method are in most cases equivalent to MIC data derived from National Committee of Clinical Laboratory Standards (NCCLS) standard procedures [6]. In this report, MIC and MBC values for TeO_3_^2- ^were consistent with literature values obtained using alternate methods [9,15].

The use of neutralizing agents in metal susceptibility testing allows for more accurate comparisons between the susceptibility of planktonic cells and biofilms to metals. Metal carry over from challenge to recovery media can affect the determination of viable cell counts and bactericidal concentrations [16]. Pertinent to this method, carry over is of particular concern when comparing biofilm and planktonic cell MBEC and MBC values. For example, planktonic cells removed *in *media containing metals will be inhibited to a different extent during recovery than biofilms removed *from *the media containing metals. In this protocol, biofilms and planktonic cultures were treated with equal amounts of a neutralizing agent at a concentration with limited toxicity to the bacterial cells. Biofilm and planktonic cells were equally susceptible to metals with long exposure times, suggesting that the neutralizing agents are equivalently effective for the treatment of both bacterial forms. The limitation of chemical neutralization will be acknowledged here. Treating metal exposed biofilms with a chelator increases the number of viable cells recovered from killing kinetics experiments relative to identical experiments that do not use a neutralizing agent (J.J. Harrison, H. Ceri and R.J. Turner, unpublished data). However, it is very likely that the metal-chelator complex is toxic to bacteria [3]. The bottom line of this procedure is that the metal-chelator is less toxic than the free metal ion.

Luria-Bertani medium is appropriate for susceptibility testing of metal oxyanions, as these compounds remain soluble in this medium. In the case of metal cations, precipitation and/or complexation of metals occurs rapidly in rich media. In this instance, minimal media preparations may be more suitable (an excellent array of minimal media preparations have been designed by Teitzel and Parsek [2]). Although the absolute values of MIC, MBC and MBEC determinations were greater in rich than in minimal medium, the tolerance of biofilms to metal toxicity remained time-dependent in both growth conditions.

Reports in the literature have suggested that biofilm tolerance to metals may be time-dependent [3,10,17]. Biofilms of *P. aeruginosa *are up to 600 times more tolerant to heavy metals than planktonic cells (in minimal media with 2 to 5 h exposure) [2,3]. Using the MBEC assay, it has been noted for *P. aeruginosa*, *Staphylococcus aureus*, and *E. coli *that biofilms and planktonic cells are equally susceptible to metal cations (in rich media with 24 h exposure) [10]. Similar time-dependent phenomena have been previously described for the killing of *E. coli *JM109 and *P. aeruginosa *ATCC 27853 biofilms by SeO_3_^2- ^and TeO_3_^2- ^[10,18]. Here this has been revaluated and this study demonstrates that the foundation of this is exposure time. Thus, the amount of time bacteria are exposed to toxic metals is an important controlled variable that contributes to measurements of bacterial susceptibility to metals. This is important both in terms of comparisons of biofilm and planktonic cell tolerance to metals as well as in evaluation of data in the literature.

## Conclusion

This high-throughput method is currently being used to elucidate a multifactorial model of metal tolerance in the bacterial biofilm [3,10,14,18,19]. The principle strength of this assay lies in the ability to rapidly screen changes in biofilm susceptibility to metals using diverse permutations of growth conditions, exposure times, and metal compounds. This type of combinatorial experimental approach would not be pragmatic using other methods.

The growth of different microorganisms may be calibrated to compare biofilms of equivalent cell density. This means that it is possible to compare susceptibility data of different strains without the concern that differences in resistance and/or tolerance are due to inequalities in growth of the biofilm populations. Lastly, exposure time influences the observed tolerance of biofilms to metals. Exposure time is thus a key consideration in the design of metal susceptibility assays.

A step-by-step protocol for this method is freely available from the authors [20].

## Methods

### Bacterial strains and media

*Escherichia coli *TG1, *E. coli *JM109 and *Pseudomonas aeruginosa *ATCC 27853 were stored at -70°C in Cryobanks™ (Prolab Diagnostics) according to the manufacturer's instructions. Bacteria were grown in Luria-Bertani media (pH 7.1, Difco) enriched with 0.001% vitamin B1 (LB + B1) or in the minimal salts vitamins glucose (MSVG, pH 7.1) of Teitzel and Parsek [2]. MSVG was specifically designed for metal susceptibility testing and minimizes the precipitation of metal cations in the growth medium. Sub-cultures and spot plates (see below) were grown on LB + B1 with 1.5% w/v granulated agar. All serial dilutions were performed using 0.9% saline.

### Biofilm cultivation

**Figure 2:**
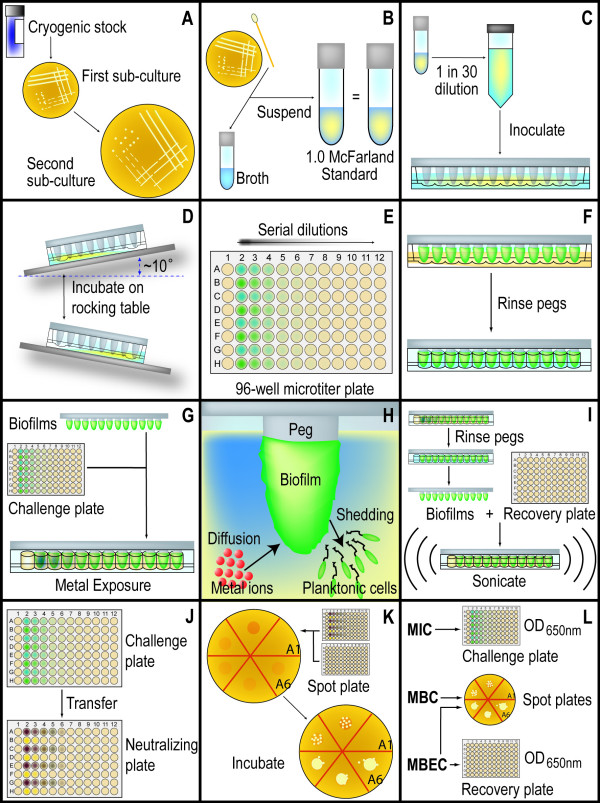
**An overview of the high-throughput protocol for metal susceptibility testing using the MBEC assay**. (A) Frozen stocks of bacteria were streaked out on the appropriate agar medium to obtain a first- and a subsequent second-subculture. (B) Colonies were collected from second-subcultures and suspended in broth medium to a 1.0 McFarland Standard. (C) This suspension was diluted 30-fold in broth, and the 1 in 30 dilution was used to inoculate the MBEC assay. (D) The inoculated device was placed on a rocking table in an incubator. (E) Serial dilutions of metal cations and oxyanions were set up along length of a microtiter plate along (the challenge plate). (F) The biofilms were rinsed to remove loosely adherent planktonic bacteria. (G) The first peg from each row was removed. These pegs were used to verify growth of the biofilms on the pegs. The peg lid was then inserted into the challenge plate. (H) During exposure, metals diffuse into the biofilm while planktonic cells are shed from the surface of the biofilm. Sloughed cells serve as the inoculum for planktonic MIC and MBC determinations. (I) The exposed biofilms were rinsed twice and the peg lid was inserted into fresh recovery medium containing the appropriate neutralizing agent (the recovery plate). The biofilms were disrupted into the recovery medium by sonciation on a water table sonicator. (J) Aliquots of planktonic cultures were transferred from the challenge plate to a microtiter plate containing the appropriate neutralizing agents (the neutralizing plate). (K) An aliquot from the recovery and neutralizing plates were spotted onto rich agar media. (L) MIC values are determined by reading the optical density at 650 nm (OD_650_) of the challenge plate after the desired period of incubation using a microtiter plate reader. Spot plates were qualitatively scored for growth to obtain MBC and MBEC values. MBEC values were redundantly determined by determining the A_650 _of the recovery plates after incubation.

The MBEC assay has two parts: 1) a plastic lid with 96 pegs that fits into 2) a corrugated trough. Biofilms were grown in the MBEC assay according to the method of Ceri and colleagues [5,6], and bacterial cultures and inocula were prepared according to the steps illustrated in Fig. 2(a–d) and as described here. Frozen stocks of bacteria were streaked out on Luria-Bertani agar to obtain a first-subculture. A single colony was picked from the first-subculture and again streaked out on agar obtain a second sub-culture. Colonies were collected from second-subcultures using a sterile cotton swab and suspended in broth medium to a 1.0 McFarland Standard. This suspension was diluted 30-fold in broth, and 22 ml of the 1 in 30 dilution was used to inoculate the MBEC assay. For demonstration purposes in this manuscript, we examined *E. coli *TG1, *E. coli *JM109 or *P. aeruginosa *ATCC 27853. Here, 22 ml of inoculum contained ~10^7 ^cfu/ml bacteria and this was transferred into the troughs. Starting bacterial number in the inocula were verified by viable cell counting. The inoculated devices were placed on a rocking table (Bellco Biotechnology) in an incubator at 35°C and 95% relative humidity at 2.5 rocks per minute. The shear force of the rocking motion facilitated the formation of 96 equivalent biofilms on the pegs.

Incubation times in LB + B1 medium were calibrated according to the growth rates of *P. aeruginosa *ATCC 27853 and *E. coli *TG1 (i.e. so that they would produce biofilms with an equivalent number of cells). Thus, *E. coli *and *P. aeruginosa *were grown for 24 and 9.5 h, respectively. For every MBEC assay, four pegs were broken from the lid (after it had been rinsed, see below), then 'sonciated' in sterile 0.9% saline. This was performed using an Aquasonic water-table sonicator (VWR International, model 250HT) for 5 minutes on the setting 'high' as previously described [5,6]. The disrupted biofilms were serially diluted and plated for viable cell counting. This growth control was used to verify that the appropriate number of bacteria had formed in the biofilm.

A test for equivalent biofilm growth on the rows of pegs in the MBEC assay was performed. This was accomplished by growing biofilms to the desired cell density, rinsing the pegs (i.e. by placing the peg lid into a microtiter plate with 200 μl of 0.9% saline in each well, termed a 'rinse plate'), then by sonicating (as described above) the biofilms into sterile saline (i.e. a fresh rinse plate). The disrupted biofilms were serially diluted then plated onto agar to determine viable cell counts (i.e. cfu peg^-1^). Plates were incubated for 24 h at 35°C then enumerated. Raw data were first log_10_-transformed and the mean viable cell counts for the different rows of pegs were compared using one-way analysis of variance (ANOVA).

### Stock metal solutions

Silver nitrate (AgNO_3_), aluminum sulfate (Al_2_(SO_4_)_3_·18H_2_O), zinc sulfate (ZnSO_4_·7H_2_O), stannous chloride (SnCl_2_·2H_2_O) and copper sulfate (CuSO_4_·5H_2_O) were obtained from Fisher Scientific Company of Fairlawn, NJ. Nickel sulfate (NiSO_4_·6H_2_O), mercuric chloride (HgCl_2_), cobalt chloride (CoCl_2_·6H_2_O), lead nitrate (Pb(NO_3_)_2_), potassium tellurite (K_2_TeO_3_) and sodium selenite (Na_2_SeO_3_) were obtained from Sigma Chemical Company of St Louis, MO. Cadmium chloride (CdCl_2_·5/2H_2_O) was purchased from Terochem Laboratories of Edmonton, AB, and manganous sulfate (MnSO_4_H_2_O) from BDH of Toronto, ON. Reagent grade metal and metalloid compounds were purchased for the purposes of this study to minimize the potential influence of contaminating, residual metals.

All stock metal solutions, with the exception of Sn^2+^, were made up in double-distilled water at 5 times the highest concentration desired in the challenge plates. These stock solutions were passed through a 0.22 μm syringe filter into sterile glass vials and stored at room temperature. Sn^2+ ^was disolved in 50% ethanol and stored in a sterile polypropylene tube.

### Metal susceptibility testing

Susceptibility testing and exposure of biofilms to metals is summarized in Fig. 2(e–l). The peg lid of the MBEC assay fits inside a standard 96-well microtiter plate. This assay may standardized to any standard brand of microplate. The volume of the challenge media in the microplate must be sufficient to submerge the peg past the height of the biofilm produced in the device. Here, we used flat-bottom 96-well microtiter plates (Nunc) with a final volume of 200 μl. Serial two-fold dilutions of metalloid oxyanions were made in LB + B1 broth along the length of these microtiter plates (termed the 'challenge plate'), allowing the first well of each row to serve as a sterility control, and the last well to serve as a growth control. The challenge plates were incubated for the desired exposure time at 35°C and 95% relative humidity. After exposure, the peg lid was removed and rinsed twice with 0.9% saline and the biofilm disrupted by sonciation into LB + B1 broth containing the appropriate neutralizing agent (termed the 'recovery plate', see below). After removal of the peg lid, the challenge plate was covered with a new, sterile lid to protect the planktonic cultures in the challenge plate wells. The planktonic cultures were also treated with the appropriate neutralizing agent (see below).

Aliquots of the neutralized biofilm and planktonic cultures were spot plated onto LB + B1 agar and incubated for 48 h at 35°C. The MIC was determined by reading the optical density of the challenge plate at 650 nm (OD_650_). MBC and MBEC values were determined by qualitatively scoring the spot plates for bacterial growth. With the exception of Cu^2+ ^and Ni^2+ ^assays, MBEC values were redundantly determined by reading the OD_650 _of the recovery plates after 48 h.

### Neutralizing agents

To differentiate between the bacteriostatic and bactericidal actions of the tested compounds, a two-step neutralizing protocol was designed to reduce the toxicity of residual metals. First, metal cations and oxyanions were treated with a chemical known to chelate or to react with the tested compound. The neutralizing agents currently and previously used in our laboratories have been summarized in Table 4. Second, neutralized cultures were plated onto a rich agar medium. Ideally, this latter step facilitates additional complexation of metals with components of the medium (such as phosphates, sulfates, and amines), and also allows diffusion of the metals into the agar. Thus, exposed bacteria are left on top of the agar medium to recover where there is a reduced concentration of biologically available metal.

As examples encountered in this study, metalloid oxyanions were reacted with 5 mM reduced glutathione (GSH, Sigma Chemical). GSH is used by the bacterial cell as a reduction-oxidation buffer to reductively eliminate a diverse array of inorganic oxidants [21,22], including selenite [23] and tellurite [24], and this is the basis for its use as a neutralizing agent here. Similarily, GSH was used to counter the effects of Zn^2+^, Co^2+^, Pb^2+^, Hg^2+^, and Cd^2+ ^toxicity. Many metals are postulated to exert toxicity through oxidative stress on the thiol groups of proteins [25,26] and thus addition of GSH or L-cysteine may partially counteract this mechanism [16]. Sodium diethyldithiocarbate (Na_2_DDTC) was used to chelate Ni^2+ ^and Cu^2+^, which rapidly formed metal precipitates with this organic chelator [27]. Citrate was used to coordinate Ag^+ ^[10]. Tin was complexed using the amino acid glycine [28]. We have previously reported that 5-sulfosalicylic acid may be used as a chelator of Al^3+ ^and Mn^2+ ^[10]. As an improvement to this technique, we suggest that crushed asprin may be used as an alternative. Salicylate derivatives can be toxic to bacteria. The less soluble acetylated form may be employed with a wider range of bacterial strains (J.J. Harrison, H. Ceri, and R.J. Turner, unpublished data).

Stock solutions of citrate (0.5 M, Sigma), Na_2_DDTC (0.25 M, ICN), glutathione (0.25 M, Sigma), acetylsalicylic acid (~0.01 M, West-Can Pharmaceuticals, available at Calgary Coop), glycine (0.25 M, Bio-Rad Laboratories), and L-cysteine (0.25 M, Sigma) were prepared in double-distilled water and sterile filtered. With the exception of acetylsalicylic acid (ASA), all of these stocks were stored at -20°C until use. ASA was stored at room temperature. Neutralizing agents for biofilm cultures were added directly to LB+B1 broth used in the recovery plates. Neutralizing agents for the planktonic cultures were prepared at 5 times the desired neutralizing concentration in 0.9% saline. Aliquots (10 μl) of the diluted stock solutions were then added to the wells of a sterile 96-well plate (the neutralizing plate) to which 40 μl from each well of the challenge plate were added. The final concentration of neutralizing agent used to treat the planktonic cultures was thus equal to that used to treat biofilm cultures.

### Scanning electron microscopy

Biofilms were examined using scanning electron microscopy (SEM) as previously described [4,6,10]. Briefly, pegs were broken from the lid of the MBEC device, rinsed once in 0.9% saline, then fixed for 16 h at 4°C in 5% glutaraldehyde dissolved in 0.1 M cacodylate buffer (pH 7.2). The next day, pegs were rinsed with 0.1 M cacodylate buffer, dehydrated with 95% ethanol, and then air dried for 30 h before mounting. SEM was performed using a Hitachi model 450 scanning electron microscopy according to the method of Morck and colleagues [29].

### Statistical tests

Mean, standard deviation and one-way analysis of variance (ANOVA) calculations were performed using GraphPad InStat 3.0 (GraphPad Software Inc., San Diego, CA).

## List of abbreviations

CLSM = confocal laser-scanning microscopy, MBC = minimum bactericidal concentration, MBEC = minimum biofilm eradication concentration, MIC = minimum inhibitory concentration, Na_2_DDTC = sodium diethyldithiocarbamate

## Authors' contributions

JJH carried out the studies of metal susceptibility, designed the experimental protocols, performed statistical analysis of the data, and drafted the manuscript. HC and RJT conceived of the experiments, participated in their design, and helped to draft the manuscript. All authors read and approved the final manuscript.

## Supplementary Material

Additional File 1 **HTP Metal Testing Protocol.doc - A step-by-step protocol**. This is a copy of the step-by-step protocol to be made available free of charge on the world wide web through the authors' web site or BMC Microbiology whichever is preferred. Click here for file

## Figures and Tables

**Table 1:** Comparative susceptibility of bacterial biofilms to metalloid oxyanions with 4 hours of exposure

Strain	Metal	MIC (mM)	MBC (mM)	MBEC (mM)	Fold tolerance^1^
*E. coli *TG1	SeO_3_^2-^	> 5.8	> 5.8	> 5.8	na
	TeO_3_^2-^	0.02 ± 0.01	0.03 ± 0.02	> 2.0	≥ 133
*P. aeruginosa *ATCC 27853	SeO_3_^2-^	> 187	> 187	> 187	na
	TeO_3_^2-^	1.6 ± 0.8	6.6 ± 3.0	> 16	2.4

na indicates a measurement that is not applicable

^1^the fold tolerance, given the sensitivity of the assay on a log_2 _scale, is equal to the ratio of the means of MBEC:MBC

**Table 2:** Susceptibility of *Escherichia coli *JM109 to metal cations with 2 or 24 h of exposure in rich (LB + B1) medium

Periodic group	Metal ion	Exposure time (h)	MIC (mM)	MBC (mM)	MBEC (mM)	Fold Tolerance^1^
7B	Mn^2+^	2	37 ± 0	> 149	> 149	na
		**24**		198 ± 86	223 ± 86	**1.1**
8B	Ni^2+^	2	7.5 ± 2.1	> 140	>140	na
		**24**		17 ± 0	29 ± 11	**1.7**
1B	Cu^2+^	2	4.5 ± 1.4	16 ± 0	16 ± 0	1.0
		**24**		16 ± 0	16 ± 0	**1.0**
	Ag^+^	2	0.06 ± 0.02	0.06 ± 0.02	1.6 ± 0.7	24
		**24**		0.06 ± 0.02	0.04 ± 0	**0.7**
2B	Zn^2+^	2	4.2 ± 2.4	125 ± 0	> 125	≥ 2.0
		**24**		31 ± 0	31 ± 0	**1.0**
	Cd^2+^	2	1.1 ± 0.2	55 ± 21	73 ± 0	1.3
		**24**		2.3 ± 0	3.0 ± 1.3	**1.3**
	Hg^2+^	2	0.04 ± 0.02	0.14 ± 0.04	0.32 ± 0.22	2.3
		**24**		0.04 ± 0	0.04 ± 0	**1.0**
3A	Al^3+^	2	nd	> 304	> 304	na
		**24**		19 ± 0	19 ± 0	**1.0**
4A	Sn^2+^	2	nd	> 17	> 17	na
		**24**		17 ± 0	17 ± 0	**1.0**

A portion of the data in this table (at the 24 h timepoint) represents a greater number of trials and reanalysis of the data originally reported in an earlier study [10].

na indicates a calculation that is not applicable

nd indicates an MIC that could not be determined due to metal precipitation

**bold **indicates the fold tolerance at 24 h of exposure

^1^the fold tolerance, given the sensitivity of the assay on a log_2 _scale, is equal to the ratio of the means of MBEC:MBC_100_

**Table 3:** Susceptibility of *Escherichia coli *JM109 to metal cations with 2 or 24 h of exposure in minimal (MSVG) medium

Periodic group	Metal ion	Exposure time (h)	MIC (mM)	MBC (mM)	MBEC (mM)	Fold Tolerance^1^
7B	Mn^2+^	2	33 ± 9	> 149	> 149	na
		**24**		93 ± 37	84 ± 47	**0.90**
8B	Co^2+^	2	≤ 0.27	26 ± 10	> 139	≥ 11
		**24**		1.0 ± 3	1.0 ± 3	**1.0**
	Ni^2+^	2	≤ 0.27	> 139	> 139	na
		**24**		0.48 ± 0.13	0.54 ± 0.39	**1.1**
1B	Cu^2+^	2	≤ 0.25	≤ 0.25	≤ 0.25	na
		**24**		≤ 0.25	≤ 0.25	na
	Ag^+^	2	≤ 0.04	≤ 0.04	0.59 ± 0	≥ 15
		**24**		≤ 0.04	≤ 0.04	**1.0**
2B	Zn^2+^	2	≤ 0.25	11 ± 6	> 125	≥ 23
		**24**		0.55 ± 0.31	0.49 ± 0.35	**0.89**
	Cd^2+^	2	≤ 0.14	5.1 ± 2.9	> 73	≥ 29
		**24**		0.49 ± 0.14	1.4 ± 1.1	**2.9**
	Hg^2+^	2	≤ 0.02	≤ 0.02	0.05 ± 0.02	≥ 2.5
		**24**		≤ 0.02	≤ 0.02	**1.0**
3A	Al^3+^	2	nd	42 ± 40	> 304	≥ 14
		**24**		1.5 ± 0.6	4.0 ± 1.4	**2.7**
4A	Sn^2+^	2	2.2 ± 0	8.6 ± 0	8.6 ± 0	1.0
		**24**		4.3 ± 0	7.5 ± 2.2	**1.7**
	Pb^2+^	2	nd	20 ± 0	40 ± 0	2.0
		**24**		4.9 ± 0	1.2 ± 0	**0.25**

na indicates a measurement that is not applicable

**bold **indicates the fold tolerance at 24 h of exposure

^1^the fold tolerance, given the sensitivity of the assay on a log_2 _scale, is equal to the ratio of the means of MBEC:MBC_100_

**Table 4:** Potential neutralizing agents for the microbiological application of inactivating metals cations and oxyanions*

Metal(loid)	Neutralizing agent	Maximum concentration^1^	Reference(s)
All oxyanions^2^	Glutathione	10 mM	[10, 18, 24]
Al^3+^, Mn^2+^	Crushed acetylsalicylic acid (ASA)	~1–2 mM^3^	[30, 31]
Hg^2+^, Cd^2+^	Glutathione	10 mM	[10]
	L-cysteine	10 mM	[16]
Cu^2+^, Ni^2+^	Diethyldithiocarbamic acid^3 ^(DDTC)	2.5 mM (*E. coli*) up to 5 mM (*P. aeruginosa*)	[27]
Sn^2+^	Glycine	10 mM	[28]
Ag^+^	Sodium citrate	10 mM	[10]
Zn^2+^, Co^2+^, Pb^2+^	Glutathione	10 mM	[3]

*This is the first part of a two-part strategy to reduce the *in vitro *toxicity of metals (see text for details)

^1 ^The maximum concentration tested and employed in studies by our laboratories using the high-throughput metal susceptibility testing method presented in this paper.

^2 ^Tested heavy metal and metalloid oxyanions from our laboratories include TeO_3_^2-^, TeO_4_^2-^, SeO_3_^2-^, CrO_4_^2-^, AsO_4_^3-^, AsO_2_^-^, WO_4_^2- ^and MoO_4_^2-^.

^3 ^Application is limited by the low solubility of salicylic acid and its acetylated derivatives in water.

^4 ^The maximum concentration listed is inhibitory to bacterial growth in broth culture. Recovery broth media must be spot plated onto agar to allow bacterial growth and determination of accurate MBC and MBEC values.
