# Supplementary material for: High-throughput metal susceptibility testing of microbial biofilms
Source: BMC Microbiol. 2005 Oct 3;5:53. doi: 10.1186/1471-2180-5-53 (PMC1262724; doi:10.1186/1471-2180-5-53)
Supplement: Additional File 1 — HTP Metal Testing Protocol.doc - A step-by-step protocol. This is a copy of the step-by-step protocol to be made available free of charge on the world wide web through the authors' web site or BMC Microbiology whichever is preferred. [file 1471-2180-5-53-S1.pdf]

PROTOCOL

High-throughput (HTP) metal susceptibility testing of microbial biofilms using the MBEC™-HTP assay

Last revised by Joe J. Harrison, February 11, 2005.

Disclaimer: Mention of trade names or commercial products in this protocol is solely for the purpose of providing specific information and does not imply endorsement by the authors.

An overview of all steps in this protocol is provided in supplementary Figure S1.

This protocol has been developed for use with Nunc® Brand, flat bottom, 96-well microtiter plates. These microplates have a maximum volume of 300 μl per well. The medium and buffer volumes listed here may need to be adjusted for different brands of microtiter plates.

A. Inoculating the MBEC™-HTP assay plate

1. From the cryogenic stock (at -70ºC), streak out a first sub-culture of the desired bacterial strain on an appropriate agar plate. Incubate for up to 24 h at the optimum growth temperature of the microorganism. The first sub-culture may be wrapped with Parafilm™ and stored at 4ºC for up to 14 days.

2. Check the first sub-culture for purity (ie. only a single colony morphology should be present on the plate).

3. From the first sub-culture, streak out a second sub-culture on an appropriate agar plate. Incubate for up to 24 h at optimum growth temperature of the microorganism. The second sub-culture must be used within 18 to 30 h starting from the time it was first placed in the incubator.

4. Verify the purity of the second sub-culture.

IMPORTANT: DO NOT GROW THE SUB-CULTURES ON MEDIA CONTAINING A SELECTIVE AGENT. ANTIBIOTICS AND OTHER ANTIMICROBIALS MAY TRIGGER AN ADAPTIVE STRESS RESPONSE IN BACTERIA. THIS MAY RESULT IN AN ABERRANT SUSCEPTIBILITY DETERMINATION.

The following steps must be carried out in a biological safety cabinet (BSC):

5. Obtain a sterile 96-well microtiter plate. For each MBEC™-HTP assay, fill 4 ‘columns’ of the microtitre plate from ‘rows’ A to F with 180 µl of a physiological saline solution (ie. 0.9% saline or phosphate buffered saline).

6. Put 1.5 ml (plus 1.0 ml for each additional MBEC™ device being inoculated at the same time) of the desired broth growth medium into a sterile glass test tube.

7. Using a sterile cotton swab, collect the bacterial colonies on the surface of the second agar sub-culture. Cover the tip of the cotton swab with a thin layer of bacteria.

8. Dip the cotton swab into the broth to suspend the bacteria. The goal is to create a suspension that matches a 1.0 McFarland standard (ie. 3.0 x 10^8 cfu/ml). Be careful not to get ‘clumps’ of bacteria in the solution.

9. Repeat steps 6 and 7 as many times as required to match the optical standard.

10. Put 29 ml of the appropriate broth growth medium into a sterile 50 ml polypropylene tube. To this, add 1.0 ml of the 1.0 McFarland standard bacterial suspension. This 30 fold dilution of the 1.0 McFarland standard (ie. 1.0×10^7 cfu/ml) serves as the inoculum for the MBEC™ device.

11. Open the sterile package of the MBEC™-HTP assay. Put 22.0 ml of the inoculum into the corrugated trough of the MBEC™ device. Place the peg lid onto the trough. Label the device appropriately.

12. Place the device on a rocking table in a humidified incubator at the appropriate temperature. The channels in the trough of the MBEC™ device must be positioned parallel to the direction of motion.

IT IS CRITICAL THAT THE ANGLE OF THE ROCKING TABLE BE SET BETWEEN 9º AND 16º OF INCLINATION. THIS MOTION MUST BE SYMMETRICAL.

13. Place a 20 µl aliquot of the inoculum in 'row' A of each of 4 'columns' in the microtitre plate set up in step 5. Serially dilute the inoculum in 10 fold increments along the length of the microtiter plate.

14. Spot plate the serial 10 fold dilutions of the inoculum from 10^{-6} to 10^{-1} on an appropriately labeled series of agar plates. Incubate the spot plates for an appropriate period of time and score for growth. These plates are controls used to verify the starting cell number in the inoculum.

<u>B. Setting up the 'challenge' plate</u>

1. Get a brand new, sterile microtitre plate and open in it in the laminar flow hood. DO NOT USE RECYCLED MICROTITRE PLATES FOR SETTING UP CHALLENGE PLATES. For example, the Anprolene® (ethylene dioxide) gas sterilization method used to recycle the microtitre plates may discolor the plastic, and this may affect optical density measurements obtained using the microtitre plate reader.

2. Setup a working solution of metal cation or oxyanion in the appropriate growth medium. Do not dilute the growth medium by more than 20% (ie. no more than 1 part metal solution per 4 parts of growth medium). The working solution of the metal should be made at a concentration equal to the highest concentration to be tested in the challenge plate.

3. Add 200 µl of growth medium to 'column' 1 and 'column' 12 of the challenge plate. These will serve as sterility and growth controls, respectively.

4. Add 100 µl of growth medium to 'columns' 3 to 11 of the microtitre plate.

5. Add 200 µl of the working solution to 'column' 2 of the microtitre plate.

6. Add 100 µl of the working solution to 'column' 3 and 'column' 4 of the microtitre plate.

7. Using the multichannel micropipette, mix the contents of 'column' 4 by pipetting up and down. After mixing, transfer 100 µl from the wells in 'column' 4 to the corresponding wells in 'column' 5.

8. Mix and transfer 100 µl from 'column' 5 to 'column' 6. Serially repeat this mix and transfer process down the length of the microtitre plate until reaching 'column' 11.

9. Mix the contents of column 11 up and down. Extract 100 µl from each well in 'column' 11 and discard.

10. Add 100 µl of growth media to the wells in 'columns' 4 through 11.

11. Replace the lid on the challenge plate. Gently tap the plate to facilitate mixing of the metals and media.

DO NOT PREPARE CHALLENGE PLATES MORE THAN 60 MINUTES PRIOR TO USE. METALS CAN SPONTANEOUSLY REACT WITH MEDIA TO FORM REDUCTION PRODUCTS WITH ALTERED BIOLOGICAL TOXICITY.

C. Exposing the biofilms

1. Setup a sterile microtitre plate with 200 μl of physiological saline solution in every well. This plate will be used to rinse the pegs to remove loosely adherent planktonic cells from the biofilm (this is termed a 'rinse plate').

2. Setup a sterile microtitre plate with 200 μl of physiological saline solution in 4 'columns' of row A for each MBEC™ device inoculated (ie. 1 microtitre plate is required for every 3 MBEC™ devices). Fill rows B to F with 180 μl of physiological saline solution. In a second microtitre plate, fill 4 'columns' from rows A to H with 180 μl of physiological saline solution for each MBEC™ device inoculated. The first microtitre plate will be used to do serial dilutions of biofilm cultures, the second will be used to check the growth of planktonic cells in the trough of the MBEC™ device.

3. Following the desired period of incubation, remove the MBEC™-HTP device from the rocker and into the laminar flow hood. Remove the peg lid from the trough and submerse the pegs in the wells of the rinse plate. Let the rinse plate sit for 1 to 2 minutes while performing step 4 below.

4. Use a micropipette to transfer 20 μl of the planktonic culure (in the trough of the MBEC™ device) into the 180 μl of saline in row 'A' of the latter plate set up in step 2 (immediately above). Repeat this three more times for a total of 4 × 20 μl aliquots.

5. Take the remainder of the planktonic culture and discard it in a solution of dilute 5% bleach. Allow a minimum of 25 minutes to completely eradicate the culture. Immediately discard the trough in the autoclave garbage.

6. In the laminar flow hood, dip a pair of pliers into 95% ethanol. Flame the pliers using the ethanol lamp in the hood.

CAUTION: DO NOT LIGHT THE ETHANOL LAMP AND DO NOT FLAME THE PLIERS BEFORE YOUR GLOVES HAVE DRIED FOLLOWING DISINFECTION USING 70% ETHANOL.

7. Using the flamed pliers, break off pegs A1, C1, E1 and G1 from the lid of the MBEC™ device and immerse them in the 200 μl of saline in row A (and each in a different 'column') of the first plate setup in step 2.

8. Using the flamed pliers, break off pegs B1, D1, F1 and H1 and discard.

9. Insert the peg lid of the MBEC™-HTP device into the challenge plate. Place the challenge plate in the same incubator where the biofilms were formed (ie. the incubator that houses the rocking table).

10. Place the microtitre plate containing the broken pegs in the tray of the water table sonicator (Aquasonic). Sonicate on the setting 'high' for 5 minutes.

11. Serially dilute 20 μl aliquots of the planktonic cultures (from step 4) in the wells of the corresponding microtitre plate. Once sonication is complete, repeat this serial dilution process with the biofilm cultures.

12. Spot plate the serial 10 fold dilutions of the planktonic and biofilm cultures from 10^{-8} to 10^{-3} and 10^{-5} to 10^{0} on an appropriately labeled series of agar plates. Incubate the spot plates for an appropriate period of time and score for growth.

D. Neutralization plates, recovery media, MBC and MBEC determinations

Neutralization is a two part procedure. First, an inorganic agent or a chelator is used to precipitate, coordinate, or reduce the metal to a less biologically toxic species. Second, the neutralized cultures are spot plated onto rich agar media. This latter step allows the diffusion of metals into the rich agar media (where they may be coordinated or precipitated) whilst bacteria remain on top where they may recover.

Neutralizing agents should be prepared as stock solutions in the range of 0.1 to 0.5 M each. The stock solutions should be syringe filtered and stored at -20°C until use.

1. Add the appropriate neutralizing agent (in a quantity to obtain the desired concentration) to 25 ml of rich media (ex. Luria-Bertani media, Tryptic Soy Broth, etc.). 20 ml of recovery medium will be required for each MBEC™-HTP assay used. Add 200 μl of this recovery medium to each well of a brand new, 96-well microtitre plate. This plate is termed the 'recovery plate'.

2. Add the appropriate neutralizing agent (in a quantity to obtain **5 times** the desired concentration) to 5 ml of physiological saline solution. 2.0 ml of 5 × neutralizing solution will be required for each MBEC™-HTP assay used. Add 10 μl of this neutralizing solution to each well of a (preferably recycled) 96-well microtitre plate. This plate is termed the 'neutralizing plate'.

3. Prepare 2 rinse plates for every MBEC™-HTP assay used (as described in part C, step 1).

4. Remove the challenge plate from the incubator and into the laminar flow hood. Remove the peg lid and immerse in the pegs in the physiological saline of a rinse plate. Cover the challenge plate with the sterile lid of the rinse plate. The challenge plate now contains the planktonic cultures that will be used for MIC and MBC determinations. Label the planktonic cultures appropriately.

5. After approximately 1 min, transfer the peg lid from the first rinse plate into the second rinse plate. While the rinse steps are in progress, proceed with step 6.

6. Using the multichannel pipette, transfer 40 μl of the planktonic cultures from the wells of the challenge plate to the corresponding wells of the neutralizing plate. Allow a minimum of 15 minutes for the neutralization reaction to occur before spot plating.

7. Transfer the peg lid from the second rinse plate into the recovery plate setup in part D, step 1. Transfer the recovery plate (containing the pegs of the MBEC™ device into the tray of the water table sonicator. Sonicate on high for 5 min.

8. After sonication, remove the peg lid from the recovery plate and replace the original lid of the microtire plate. Note any reduction or colour changes to the biofilms on the pegs. The lid of the MBEC™ device may now be discarded.

9. Transfer 40 μl from each well of the recovery plate into the corresponding well of a sterile (preferably recycled) 96-well microtitre plate. Place the recovery plate in the same incubator used to form the biofilms and incubate a minimum of 48 h.

10. On an appropriately labeled stack of agar plates, spot 20 μl aliquots of the neutralized planktonic and biofilm cultures (use the aliquots of recovery media prepared in step 9 above, do not spot plate directly from the recovery plate). Incubate at an optimal growth temperature for a minimum of 48 h before scoring qualitatively for growth.

Log-Killing and Viable Cell Counts

Instead of preparing neutralization plates as outlined above, transfer planktonic cultures into serial dilution microtitre plates that contain 180 μl of physiological saline solution in each well of rows B to H, and 10 μl of the 5 $\times$ neutralizing solution in row A. Serially dilute 20 μl using the multichannel pipette.

Similarly for biofilm cultures, add 40 μl of the recovery media (containing the sonicated biofilms) from the recovery plate to row A of a serial dilution plate containing 180 μl of physiological saline solution in each well of rows B to F. Serially dilute 20 μl using the multichannel pipette.

Spot plate biofilm and planktonic cultures (which have been serially diluted) on appropriately labeled agar plates. Incubate for a minimum of 48 h.

To calculate log-kill, use the following formula:

$$\text{log-kill} = \log_{10}(\text{initial cfu/ml}) - \log_{10}(\text{remaining cfu/ml after exposure})$$

To calculate percent kill, use the following formula:

$$\%\ \text{kill} = 100 - \{[(\text{initial cfu/ml}) \times (\text{remaining cfu/ml}) / (\text{initial cfu/ml})] \times 100\}$$

Collecting Data

1. MIC values are obtained by reading the optical density of the challenge plate at 650 nm (OD_{650}) 48 to 72 h after the pegs have been removed from the challenge media.

2. MBC and MBEC values are determined by +/- scoring of growth on the spot plates after a minimum of 48 h incubation. (Note that after this period of time, scoring is not a time sensitive operation). Alternatively, if viable cell counts are being determined, enumerate the bacteria growing on the spot plates.

3. MBEC values are redundantly determined by reading the OD_{650} of the recovery plate on the microtitre plate reader after 48 h incubation.

Abbreviations used

MIC = *minimum inhibitory concentration;* MBC = *minimum bactericidal concentration;* MBEC = *minimum biofilm eradciation concentration;* HTP = *high-throughput;* MBEC™ = *the MBEC™-device.*

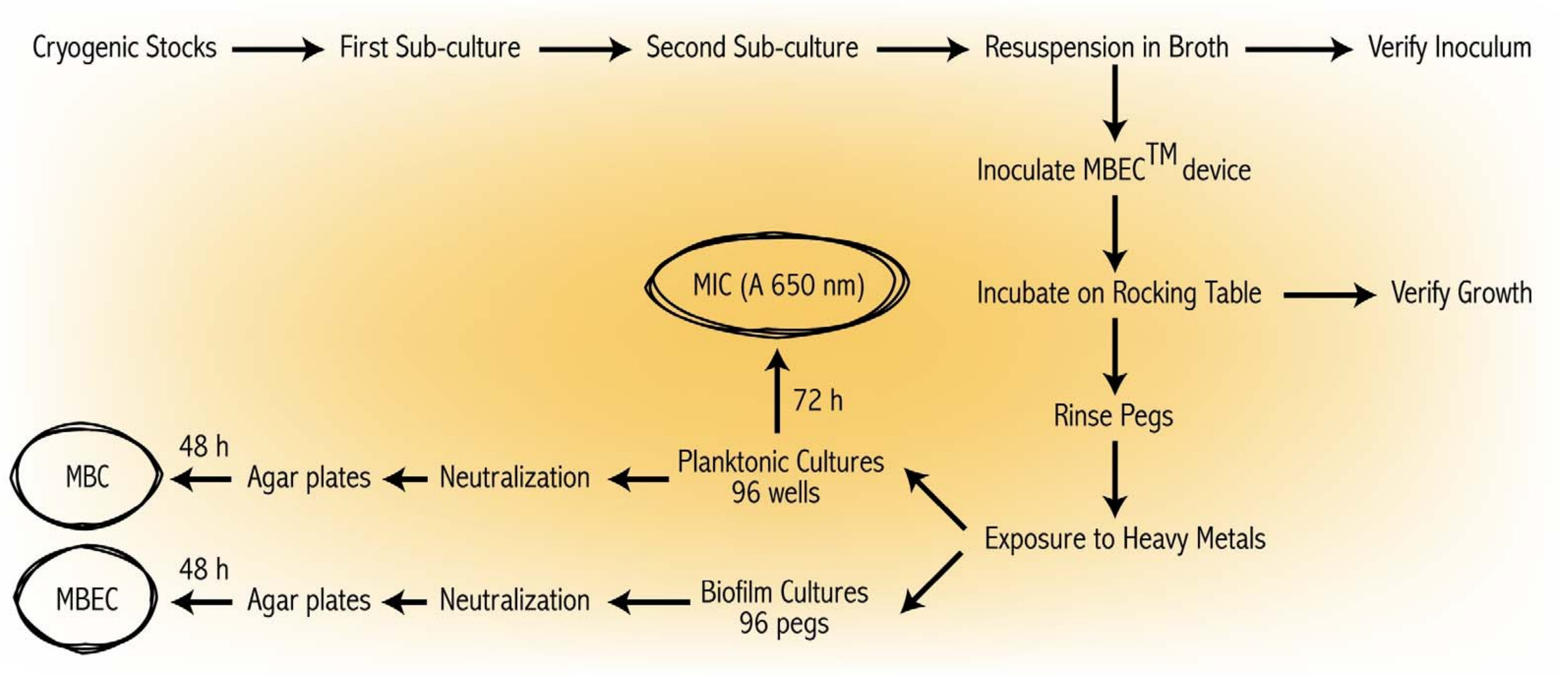

Figure S1. An overview of the high-throughput protocol for metal susceptibility testing using the MBEC™-HTP assay. Frozen stocks of bacteria are streaked out on the appropriate media to obtain first sub-cultures. A single colony is picked from the first-subculture, and streaked out on agar media again to give a second sub-culture. Colonies from the second sub-culture are resuspended in broth to a 1.0 McFarland Standard. This suspension is diluted 30-fold in broth, and 22 ml of the 1 in 30 dilution is used to inoculate the MBEC™-HTP assay. The MBEC™ device is then placed on a rocking table in an incubator. The shear force from the rocking motion facilitates the formation of 96 biofilms on the pegs of the MBEC™ device. The biofilms are rinsed, then placed in a microtitre plate containing serial dilutions of metals. Following exposure, planktonic and biofilm cultures are treated with neutralizing agents to eliminate carry-over of the metal to the recovery medium. Neutralized cultures are then spot plated onto rich agar, incubated for a minimum of 48 h, then qualitatively scored for growth (to give MBC and MBEC values). MIC values are determined by reading the optical density at 650 nm of the challenge plate after 72 h incubation using a microtitre plate reader.
